# Mechanically induced alterations in chromatin architecture guide the balance between cell plasticity and mechanical memory

**DOI:** 10.3389/fcell.2023.1084759

**Published:** 2023-04-18

**Authors:** Adrienne K. Scott, Michael Rafuse, Corey P. Neu

**Affiliations:** ^1^ Paul M. Rady Department of Mechanical Engineering, University of Colorado Boulder, Boulder, CO, United States; ^2^ Biomedical Engineering Program, University of Colorado Boulder, Boulder, CO, United States; ^3^ BioFrontiers Institute, University of Colorado Boulder, Boulder, CO, United States

**Keywords:** cellular plasticity, mechanotransduction, mechanical memory, chromatin, epigenetics

## Abstract

Phenotypic plasticity, or adaptability, of a cell determines its ability to survive and function within changing cellular environments. Changes in the mechanical environment, ranging from stiffness of the extracellular matrix (ECM) to physical stress such as tension, compression, and shear, are critical environmental cues that influence phenotypic plasticity and stability. Furthermore, an exposure to a prior mechanical signal has been demonstrated to play a fundamental role in modulating phenotypic changes that persist even after the mechanical stimulus is removed, creating stable mechanical memories. In this mini review, our objective is to highlight how the mechanical environment alters both phenotypic plasticity and stable memories through changes in chromatin architecture, mainly focusing on examples in cardiac tissue. We first explore how cell phenotypic plasticity is modulated in response to changes in the mechanical environment, and then connect the changes in phenotypic plasticity to changes in chromatin architecture that reflect short-term and long-term memories. Finally, we discuss how elucidating the mechanisms behind mechanically induced chromatin architecture that lead to cell adaptations and retention of stable mechanical memories could uncover treatment methods to prevent mal-adaptive permanent disease states.

## Introduction

Phenotypic plasticity in response to external signals is crucial for a cell to survive and maintain homeostasis. Phenotypic plasticity is defined as the ability of cells to adapt (e.g., changes in morphology, physiology, or behavior) in response to intrinsic or external cues (Nemec and Kilian, 2020). A cell’s adaptation relies upon a myriad of mechanical and biochemical stimuli, resulting in the activation or repression of numerous gene expression profiles that lead to cellular remodeling. At the same time, cells must maintain developmental stability, or a phenotype consistent to the developmental trajectory specific to the cell type, to carry out specific roles in the human body. Hence, the balance between stability and phenotypic plasticity is vital to maintaining a functioning organ system.

The mechanical plasticity of the cell is largely a function of how the cell senses and responds to mechanical signals through mechanotransduction pathways (Lammerding et al., 2004a). Mechanotransduction pathways are stimulated by many external physical cues such as matrix stiffness and tensile, compressive, or shear stress. Membrane level proteins, such as stretch activated ion channels (e.g., Piezo, TRP, TREK/TRACK channels) (Jin et al., 2020), focal adhesion complexes (e.g., focal adhesion kinase, FAK), and cell-cell junctions (e.g., cadherins) are sensors that initiate cascading pathways resulting in changes in transcription in the nucleus. For example, cells sense increased stiffness of the environment through focal adhesions which initiates signaling cascades, such as Rho/ROCK, PI3K/AKT, and MAPK, resulting in changes in gene expression through activation of transcription factors (Martino et al., 2018). Examples of well-studied mechanosensitive molecules regulating transcription include the serum response factor (SRF) and the yes associated protein (YAP) (Dupont et al., 2011). These and other mechanotransduction pathways have been extensively explored and reviewed (Jaalouk and Lammerding, 2009; Martino et al., 2018; Wagh et al., 2021; Zuela-Sopilniak and Lammerding, 2022). Forces applied to the membrane can also directly influence the genetic programs that dictate the phenotypic response of the cell since the cytoskeletal network is linked to the nuclear structure containing chromatin and DNA through the linker of nucleoskeleton and cytoskeleton (LINC) complex (Lombardi et al., 2011), which also has been reviewed extensively (Lombardi and Lammerding, 2011; Bouzid et al., 2019; Lityagina and Dobreva, 2021; Wong et al., 2021). Although the mechanisms of mechanotransduction that mediate phenotypic plasticity have been well characterized in many systems, what is less known is how the mechanical environment alters cell fate and establishes a stable response, or memory.

Recent literature shows that alterations in the mechanical environment can lead to stable responses that persist even after the mechanical stimulus is removed, a phenomenon called mechanical memory (Kanoldt et al., 2018; Dai et al., 2020). Although the mechanisms of mechanical memory remain largely unexplored, recent studies focus on how changes in chromatin architecture retain responses to the mechanical environment altering the long-term cell fate (Heo et al., 2015; Killaars et al., 2019; Walker et al., 2021). By using cardiac physiology and pathology as an example, this mini review explores how the mechanical environment influences phenotypic plasticity and stable mechanical memories, specifically by altering the chromatin architecture of cells.

### Mechanical plasticity for maintaining organ-level and cell-level homeostasis

Often the plastic response of a cell allows for beneficial adaptations that lead to maintaining physiological homeostasis. When there is a mechanical perturbation that disrupts this stability, the cell will adapt to this change in an attempt to return the system to a mechanically stable state, or mechanostasis (Chan et al., 2011). The heart is a highly plastic organ that demonstrates the transition between alternate stable states. In cases of increased hemodynamic load (such as pregnancy, repeated exercise, or postnatal growth) the heart muscle will undergo hypertrophic growth to accommodate the new output requirements (Tingare et al., 2013). The left ventricle of well-trained athletes can exceed the mass of non-athletes by as much as 60% (Hill and Olson, 2008). Despite this tremendous alteration, the cells maintain their plasticity; the physiological hypertrophy is reversible when the increased demand is removed. The end of an intensive training schedule leads to cardiac atrophy of 10%–22% in humans (Perhonen et al., 2001). This is also observed after pregnancy when a woman’s heart will decrease in size and return to the pre-pregnancy mass (Tingare et al., 2013).

These highly plastic responses are enabled by cellular proteins that sense the changes in mechanical cues and adapt accordingly. In the case of cardiac muscle size and function, the plastic response arises from remodeling of cardiac cells and the surrounding ECM. For example, increased mechanical loading due to pressure overload has been shown to cause sarcomere deposition in parallel, leading to the increase in myocyte cross-sectional area ultimately causing concentric hypertrophy. In contrast, volume overload often results in the elongation of myocytes due to the deposition of sarcomeres in series, which leads to dilation of the ventricle (Lammerding et al., 2004a; Lyon et al., 2015). Specifically, a change in the mechanical stretch is sensed by many mechanosensors in cardiac tissue: stretch activated ion channels (Yamazaki et al., 1998; Reed et al., 2014), integrins and integrin associated proteins (melusin, integrin-linked kinase) (White et al., 2006), cell surface receptors (G-protein-coupled receptors and angiotensin II type receptors), cytoskeletal and sarcomeric proteins (titin, myosin, or the small LIM domain protein MLP) (Lyon et al., 2015), and the nucleus (Lammerding et al., 2004a). Ultimately, the mechanical signal alters the cardiomyocyte phenotype via changes in gene expression resulting from changes in calcium concentration and activation of transcription pathways such as MAPK, JAK/STAT, PKC, PI3K, and Hippo-YAP/TAZ (Ruwhof and van der Laarse, 2000; Lammerding et al., 2004a; Saucerman et al., 2019). The focal adhesion-integrin complex is a main mechanosensor in cardiac fibroblasts and is known to activate many pathways leading to the production of ECM and fibrosis, including MAPK and p38 pathways (Davis and Molkentin, 2014; Saucerman et al., 2019). In addition to the activation of transcription factors, epigenetic mechanisms that regulate gene transcription could also provide another layer of gene transcription regulation by external mechanical cues (Saucerman et al., 2019).

### Epigenetic landscape: Chromatin architecture changes in plastic verses stable responses

Epigenetic regulation, or the processes involving chemical modifications of chromatin that influence gene transcription, allows for both phenotypic plasticity in response to environmental cues and the formation of stable memories. Epigenetic mechanisms, such as histone modifications or DNA methylation, are reversible (although DNA methylation is typically more stable than histone modifications) (Tzelepis et al., 2017). At the same time, epigenetic mechanisms can be inherited through cell division, thus maintaining a stable epigenetic memory across generations (Henikoff and Greally, 2016; Kim and Costello, 2017). On a single gene level, the epigenetic modifications along with the spatial organization of chromatin by architectural proteins, give rise to the overall 3-dimensional (3D) chromatin architecture. The spatial organization of chromosomes is dynamic and adaptable to environmental changes (Solovei et al., 2009; Uhler and Shivashankar, 2017) and has been shown to be altered in disease states (Seelbinder et al., 2021; Walker et al., 2021; Heo et al., 2022). However, 3D architectures are also known to be cell type specific and thus demonstrate developmental stability (Seelbinder et al., 2021; Winick-Ng et al., 2021). Therefore, the cellular epigenetic regulation and alterations in chromatin architecture that determine the epigenetic landscape can control both the stability and phenotypic plasticity of cells.

**FIGURE 1:**
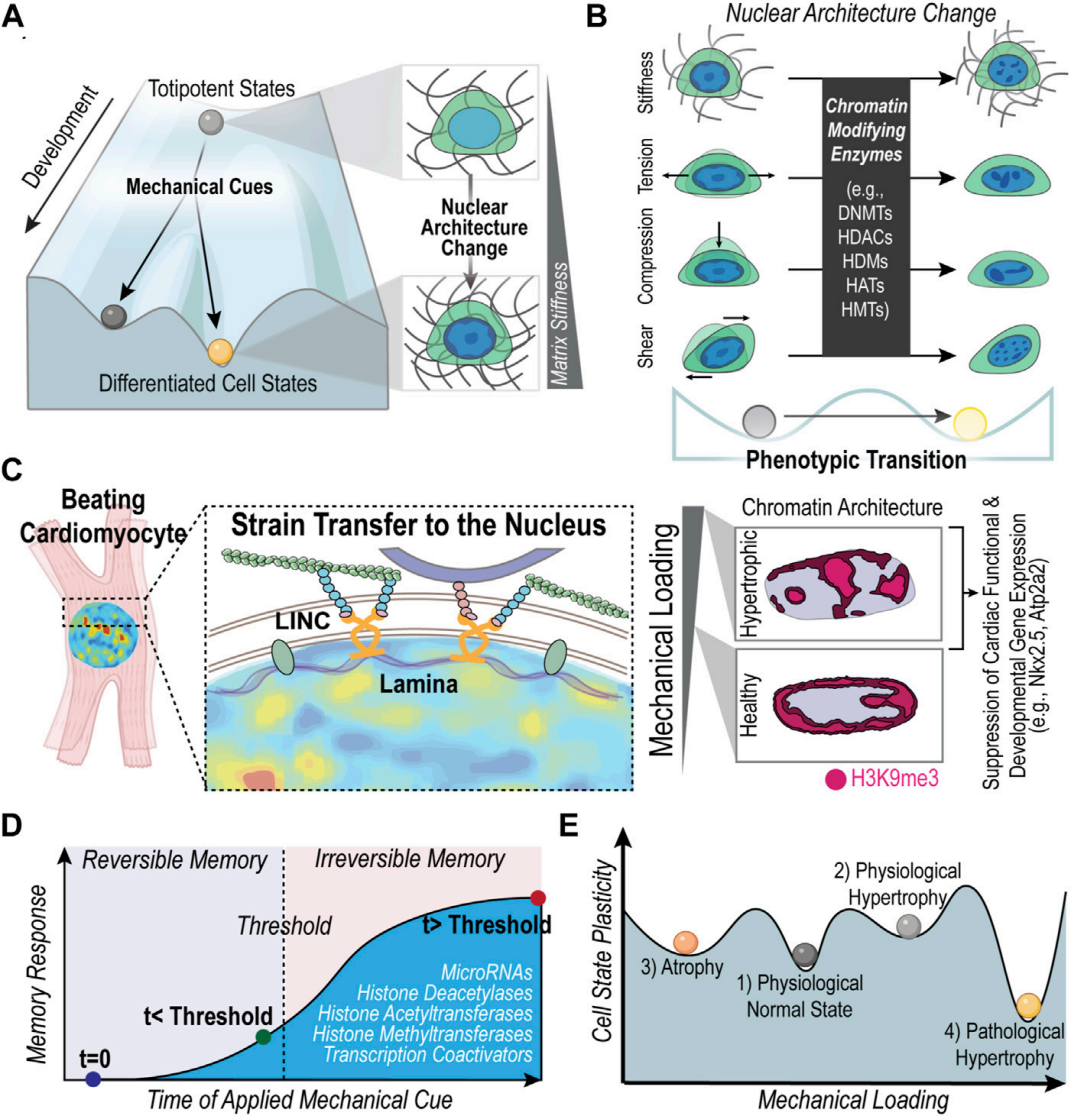
Mechanically induced nuclear architecture changes along Waddington’s landscape of epigenetic regulation. **(A)** The action of balls rolling downhill represents the increasing epigenetic changes that restrict the genetic landscape and ultimately lead to stable phenotypes. Environmental cues, such as the mechanical environment, can dictate cell fate changes and the overall differentiation process. As cells differentiate, the chromatin architecture changes (Peric-Hupkes et al., 2010; Zheng and Xie, 2019) and reflects a different state on the epigenetic landscape. **(B)** Changes in epigenetics due to mechanical cues (e.g., matrix mechanical properties, compression, tension, and shear forces) also alter the phenotypic state of cells, demonstrating that epigenetic regulation contributes to both the developmental stability and phenotypic plasticity of cells (Tzelepis et al., 2017). Chromatin architecture changes are also known to indicate a phenotypic change, especially in disease states (Seelbinder et al., 2021; Walker et al., 2021; Heo et al., 2022). **(C)** Previous work has demonstrated how a pathologically stiff mechanical environment in cardiac tissue is associated with a disruption of the chromatin architecture of repressive histone modification, H3K9me3, which has been linked to the dysregulation of cardiomyocyte differentiation and function (Seelbinder et al., 2021). Alterations in chromatin architecture of cardiomyocytes were associated with changes in nuclear deformation. Key molecular players that influence nuclear deformation of cardiomyocytes include nesprin proteins in the LINC complex (Ghosh et al., 2019). Literature also supports that lamin proteins are key regulators of nuclear mechanics and are associated with a loss of cardiac function (Lammerding et al., 2004b; Ghosh et al., 2019). **(D)** Cells exhibit mechanical memory; the history of mechanical priming can influence cell fate in a reversible and non-reversible manner depending on the exposure time to the mechanical cue. Molecules involved in storing mechanical memory include microRNAs, histone modifications, histone modifying enzymes and transcriptional coactivators (e.g., YAP). **(E)** As the mechanical load on heart cells increases or decreases the cell shifts to a new state of mechanostasis, shown as an equilibrium ‘valley’ on the graph. The cells in each state have different degrees of plasticity. The depth of the ‘valleys’ is proportional to the inability to adapt, or the lower degree of plasticity. For example, because the physiological hypertrophic state (2) is known to be highly reversible, its state of mechanostasis is depicted as a shallow ‘valley.’ Created with adapted graphics from BioRender.com.

Conrad Waddington proposed the metaphor of the “epigenetic landscape” to describe the changes in cell phenotypic plasticity and stability with changes in epigenetic regulation (Figure 1A). The action of balls rolling down a hill represents the accumulation of epigenetic changes during development that lead to stable differentiated cell states (Goldberg et al., 2007) (Figure 1A). At the same time, the metaphorical balls can roll into valleys adjacent to the current position. This transition represents cell phenotypic changes in response to external cues (e.g., mechanical cues), which can alter the chromatin architecture of the cell (Figures 1A, B). For example, cardiac fibroblasts will become activated into a myofibroblast state in response to a stiffening environment (Wang et al., 2003), which has been shown to change the chromatin architecture (Walker et al., 2021). The phenotypic plasticity or stable memory of the altered state of chromatin architecture is denoted by the depth of the valley in Waddington’s epigenetic landscape.

### How mechanical cues alter the epigenetic landscape and chromatin architecture

An increasing body of literature demonstrates the connections between the mechanical environment and the epigenetic landscape of cells (Spagnol et al., 2016; Miroshnikova et al., 2017; Wagh et al., 2021; Dupont and Wickström, 2022). Seminal work in the field demonstrated that substrate stiffness guides differentiation of mesenchymal stem cells (MSCs), highlighting the influence of the mechanical environment on how stem cells “roll” down the epigenetic landscape (Engler et al., 2006) (Figure 1A). In fact, chromatin condensation upon dynamic loading of MSCs was shown to be mediated by the cytoskeleton (Heo et al., 2015; Heo et al., 2016). Furthermore, the chromatin remodeling of MSCs in response to stiff culture environments occurs through the increase in histone acetyltransferase (HATs) and a decrease in histone deacetylases (HDACs) (Killaars et al., 2019; Killaars et al., 2020) (Figure 1B). Such chromatin remodeling through upregulation or downregulation of chromatin modifiers has also been observed in response to compressive forces (Damodaran et al., 2018) and hydrostatic pressure (Maki et al., 2021). In addition, chromosome positioning within the nucleus is also influenced by mechanical cues of cell geometry, which in turn affects the spatial organization of genes thereby regulating gene expression (Maharana et al., 2016; Wang et al., 2017; Alisafaei et al., 2019). Interestingly, recent studies have shown that mechanical cues can even induce cellular reprogramming via chromatin modifications. For instance, substrate topological cues, such as the presence of microgrooves, can influence the epigenetic state of fibroblasts and enhance their reprogramming to iPSCs through inducing changes in H3 acetylation and methylation (Downing et al., 2013). Also, nuclear deformation in fibroblasts through their confinement in microfluidic channels has shown to increase the efficiency of their reprogramming to neurons via decreased levels of H3K9me3 and DNA methylation (Song et al., 2022). Together, these studies demonstrate that the physical environment can alter the slope of the hill in the epigenetic landscape, either accelerating the “rolling down” of differentiating cells or allowing cells to “roll back up” the landscape in reprogramming processes (Figure 1A).

Although the mechanisms of how the epigenetic landscape is influenced by mechanical cues are not completely understood, recent studies focus on 1) indirect biochemical cues arising from the activation of mechanosensitive proteins and 2) the direct transmission of force to chromatin influencing a change in accessibility of transcription (Kirby and Lammerding, 2018; Dupont and Wickström, 2022; Kalukula et al., 2022). Deformation of stretch activated ion channels, such as Piezo1 found in the perinuclear endoplasmic reticulum, have been shown to decrease levels of H3K9me3 marked heterochromatin in response to cell stretching, while levels of H3K9me3 remained unchanged in response to stretch with Piezo1 depleted cells (Nava et al., 2020). Mechanosensitive nuclear membrane protein, Emerin, enrichment around the nuclear membrane can lead to the alteration of heterochromatin anchoring to the lamina associated domain through influencing nuclear G-actin levels (Le et al., 2016). Additionally, deformation of nuclear pores can allow nuclear shuttling of chromatin modifying enzymes, such as histone deacetylase 3 (HDAC3) from the cytoplasm (Zuela-Sopilniak and Lammerding, 2022). Alternatively, the physical links through the cytoskeleton and the LINC complex can allow for physical deformation of chromatin through nuclear strain transfer, influencing gene transcription (Lombardi et al., 2011; Lombardi and Lammerding, 2011; Neelam et al., 2015; Ghosh et al., 2017; Ghosh et al., 2019; Ghosh et al., 2021; Jones et al., 2022). For example, magnetic twisting of cell surface proteins induced transcription of a fluorescently tagged transgene depending on the direction and magnitude of the applied load (Tajik et al., 2016).

In the case of cardiac cells also, there is recent literature that highlights such mechanical regulation of the epigenetic landscape and its importance in cardiomyocyte development and function (Tingare et al., 2013; Stratton and McKinsey, 2016; Jarrell et al., 2019; Lityagina and Dobreva, 2021; Ross and Stroud, 2021; Powers and McCulloch, 2022). Connections from the cardiomyocyte nucleus to the external environment through microtubules and desmin intermediate filaments is required for the maintenance of the nuclear morphology and the loss of these connections results in aberrant gene expression and DNA damage (Heffler et al., 2020). Similarly, previous work has associated disrupted nuclear morphology with cardiac pathology (dilated cardiomyopathy) (Zhou et al., 2020) and the effects of transverse aortic constriction surgery (Karbassi et al., 2019). Cardiomyocyte nuclear morphology can be altered through both external cues and changes of internal cellular proteins, such as lamin proteins and proteins in the LINC complex, such as nesprins (Lammerding et al., 2004b; Ghosh et al., 2019) (Figure 1C). Work from our lab shows that when cardiomyocyte nuclear morphology is disrupted through culture on stiff substrates or through disruption of the LINC complex, the architecture of trimethylated H3K9 marked chromatin remodels, which we linked to dysregulation of the cardiomyocyte development and function (Seelbinder et al., 2021) (Figure 1C). Our work also supports a connection between nuclear strain and heterochromatin organization since regions of higher tensile strain in cardiomyocytes were more likely to overlap with regions of trimethylated H3K9 marked chromatin (Ghosh et al., 2019; Seelbinder et al., 2021). Additionally, stiff environments have been shown to induce changes in the chromatin architecture of cardiac interstitial cells that is linked to a persisting fibrotic phenotype (Walker et al., 2021). Key regulators of the chromatin changes of these cardiac interstitial cells included the CREB Binding Protein (Walker et al., 2022) and HDACs (Walker et al., 2021), while detachment of the nucleus from the cytoskeleton prevented the chromatin architecture changes. Taken together, these results support how the mechanical environment regulates the chromatin architecture, which in turn influences phenotypic changes of cardiac cells (Figures 1B, C).

### Mechanical memory

Recent studies have found that mechanical environments can cause phenotypic transitions that are maintained, even after the mechanical stimulus is removed demonstrating a stable mechanical memory (Figure 1D; Table 1) (Tingare et al., 2013). A critical factor observed to be an important determinant of mechanical memory is the prolonged exposure to the mechanical stimulus. For example, Balestrini et al. have shown that when lung fibroblasts are primed in a pathologically stiff culture environment for 3 weeks, they display a myofibroblast phenotype for 2 weeks after transfer to healthy soft substrates (Balestrini et al., 2012). In the reverse experiment, lung fibroblasts cultured on healthy soft substrates for 3 weeks were partially protected from myofibroblast activation after shifting to the pathologically stiff substrates (Balestrini et al., 2012). Similarly, Yang et al. showed that hMSC exposure to a relatively high stiffness of 10 kPa for 10 days before lowering the stiffness to 2 kPa, led to a permanent (>10 days) nuclear localization of RUNX2, a pre-osteogenic transcription factor, and YAP/TAZ, the transcriptional regulator of differentiation induced by ECM stiffness. Interestingly, experiments probing the mechanism of mechanical memory showed that YAP/TAZ acts as a “rheostat” that regulates the reversibility or irreversibility of the osteogenic response depending on whether the mechanical dosing time exceeded a critical memory threshold (Yang et al., 2014) (Figure 1D). Another molecule identified to play a role in preserving the fibrotic mechanical memory in rat MSCs is microRNA-21. In fact, knocking down microRNA-21 was sufficient to erase accumulated mechanical memory from stiff priming (Li et al., 2017).

**TABLE 1:** Studies exploring mechanical memory in different cell types.

Cell type	Priming event	Modulated phenotype	Mechanism explored
Human MSCs Engler et al. (2006)	Culture on soft substrates (1–3 weeks)	Persistent neurogenic phenotype	Not directly explored
Rat MSCs Li et al. (2017)	Culture on stiff/soft matrix (3 passages)	Persistence or absence of fibrosis	MicroRNA-21
Bovine MSCs Heo et al. (2015)	Strain level & number of loading events (30 s–3 h)	Degree of chromatin condensation	EZH2 methyltransferase & acto-myosin contractility
Human MSCs Yang et al. (2014)	Culture on dynamic stiff/soft substrates (1–10 days)	Osteogenic lineage decision markers	YAP/TAZ
Human MSCs Killaars et al. (2019)	Culture on dynamic stiff/soft substrates (1 or 10 days)	Persistent chromatin remodeling	Histone acetylation (HDAC1/2 & HAT1)
Rat Lung Fibroblast Balestrini et al. (2012)	Culture on stiff/soft substrates (2–3 weeks)	Myofibroblast persistence	Not directly explored
Rat Adipose Stem Cells Dunham et al. (2020)	Culture on soft substrates (2 weeks)	Pro-fibrotic phenotype	Not mediated through YAP
Hepatic Stellate Cells Fan et al. (2017)	Culture on stiff/soft matrix (7 days)	Levels of contractility	Not directly explored
Human Epithelial Non-tumorigenic Cells Nasrollahi et al. (2017)	Culture on stiff/soft matrix (1–3 days)	Collective migration	Mediated through YAP
Porcine Valvular Interstitial Cells Walker et al. (2021)	Culture on stiff/soft matrix with *in vivo* disease state (1–7 days)	Persistent fibroblast state	HDACs & Nuclear/Cytoskeleton Tension
Murine Skeletal Muscle Stem Cells Gilbert et al. (2010)	Culture on stiff plastic/soft hydrogel substrates (7 days)	Differentiated state	Not explored
Human MSCs Wei et al. (2020)	Culture on stiff/soft substrates (4–7 days)	Osteogenic differentiation	MicroRNA-21
Human Adipose Stem Cells Berger et al. (2021)	Culture on stiff/soft substrates (1 passage)	Adipogenic capacity	Nesprin-2 & Peroxisome proliferator- activated receptor gamma
Human MSCs Abdeen et al. (2016)	Culture on dynamic stiff/soft substrates (4 h)	Osteogenic state	Not directly explored
Bovine Chondrocytes Scott et al. (2023)	Culture on 2D stiff plastic prior to 3D soft hydrogel (8–16 population doublings)	Dedifferentiated chondrocyte state	H3K9me3 marked chromatin architecture

In addition to the regulation of microRNAs and transcription factors, recent studies focus on chromatin remodeling induced by histone modifications as a mechanism for retaining mechanical memory (Turner, 2002; Hathaway et al., 2012; Heo et al., 2015; Fan et al., 2017) (Figure 1D). In fact, a theoretical framework to model mechanical memory required the modulation of epigenetic plasticity due to long term mechanical priming that locks in mechanical memory (Peng et al., 2017; Mathur et al., 2020). Dynamic loading applied for 150 s to 3 h resulted in increased levels of chromatin condensation in MSCs. The increased levels of chromatin condensation only persisted in cells dynamically loaded for 3 h, while chromatin condensation of cells that were loaded for less time (less than 1 h) was reversible (Heo et al., 2015). The persistence of condensed chromatin could be prevented by using pharmacological treatments to inhibit EZH2. Similarly, work from our lab shows that remodeling of H3K9me3 marked chromatin occurs when chondrocytes are exposed to stiff substrates and is later remembered when chondrocytes are transferred to a soft 3D environment. However, the remodeling of H3K9me3 can be partially disrupted with inhibitors of epigenetic modifiers (KDM4, EZH2) (Scott et al., 2023; Scott et al., 2022). Many of these examples of mechanical memory and others (listed in Table 1) highlight how changes in chromatin architecture can persist, maintaining stable memories. Therefore, not only are the mechanically induced changes in chromatin architecture involved in the plastic response of the cell, but such changes can also persist after removal of the stimulus, contributing to the stable mechanical memory of the cell.

### Phenotypic stability of mechanically induced disease states

The effort to maintain mechanostasis in the presence of certain mechanical stimuli may lead to maladaptive responses that decrease the cell’s ability to function in the new environment. For example, a continuous excessive stress on the heart muscle, as caused by high blood pressure, often leads to thickening of the left ventricle walls of the heart, known as maladaptive cardiac hypertrophy (Dorn and Force, 2005; Shyu, 2009). Unlike the highly-reversible physiological hypertrophy, the hypertrophy induced by prolonged stress is usually followed by apoptosis or necrosis and has not been shown to be easily reversed (Jaalouk and Lammerding, 2009; Tingare et al., 2013). Regression of left ventricular remodeling due to pressure overload has been demonstrated with the removal of aortic band constrictions (Gao et al., 2005; Stansfield et al., 2007; Zhao et al., 2013; Yoshida et al., 2020) in animals as well as clinically with treatment of aortic stenosis after aortic valve replacements (Krayenbuehl et al., 1989; Matsumura et al., 2008; Hahn et al., 2013), demonstrating some degree of left ventricular remodeling plasticity. Additionally, regression of left ventricular remodeling has also been seen after weight loss (Kardassis et al., 2012). However, left ventricle dysfunction sometimes persists even after 3 years following weight loss (Alexander and Peterson, 1972). Aortic banding and de-banding studies suggest that exposure time to pressure overload may influence the regression and recovery of left ventricle remodeling (Zhao et al., 2013). Further exploration is needed to identify the conditions or mechanical threshold in which cardiac remodeling leads to heart failure (irreversible memory) and when there is potential for reversal of cardiac remodeling (reversible memory) (Figure 1D).

We propose a depiction of the cardiomyocyte response to loading in Figure 1E to demonstrate how the plastic response of cardiomyocytes changes with increased loading. Each valley in Figure 1E represents an alternate mechanically stable state, such as a physiological normal cardiomyocyte or a pathological hypertrophic cardiomyocyte. The depth of the valley is proportional to the inability to change phenotype. By exploring short and long-term changes in chromatin architecture of cardiac cells in response to various exposure times and degrees of mechanical loading, we may gain a more in-depth understanding of how plasticity or long-term disease states arise in the heart.

## Discussion

Here, we have reviewed examples of how mechanical environments influence both the phenotypic plasticity and the stability of phenotypic states through changes in chromatin architecture, but many questions remain. What determines the threshold between a temporary adaptation to the physical environment and a stable memory? Perhaps 3D gene positioning relative to the areas of repressed heterochromatin in the nuclear periphery and areas of active transcriptional machinery dictate the degree of plasticity for the expression of gene programs. Alternatively, the overall 3D architecture of persistent states could be more energetically favorable from the perspective of phase separation models of chromatin (Erdel and Rippe, 2018). To what extent does cell division influence the phenotypic plasticity of cells in response to the mechanical environment? Does cell division present a window of opportunity (Probst et al., 2009) to alter cell fate in response to the surrounding mechanical environment? Furthermore, the phenomena of mechanical memory has only been demonstrated due to the exposure time to *in vitro* cultures. Can mechanical memories develop from changing mechanical environments *in vivo*? How does mechanical memory of cell states translate to organ level memory? Multi-scale computational models (Weinberg and Mofrad, 2007; Yoshida et al., 2022) incorporating cell models of mechanical memory (Nasrollahi et al., 2017) may begin to answer this question to understand how pathologies reach a point of no return. Overall, we propose that by further exploring these questions we could provide insight into methods to prevent long-term maladaptive disease states.
